# Decrease in the Traumatic Symptoms Observed in Child Survivors within Three Years of the 2011 Japan Earthquake and Tsunami

**DOI:** 10.1371/journal.pone.0110898

**Published:** 2014-10-23

**Authors:** Masahide Usami, Yoshitaka Iwadare, Kyota Watanabe, Masaki Kodaira, Hirokage Ushijima, Tetsuya Tanaka, Maiko Harada, Hiromi Tanaka, Yoshinori Sasaki, Kazuhiko Saito

**Affiliations:** 1 Department of Child and Adolescent Psychiatry, National Center for Global Health and Medicine, Kohnodai Hospital, Chiba, Japan; 2 Department of Child Mental Health, Imperial Gift Foundation, Aiiku Hospital, Tokyo, Japan; 3 Division of Neuropsychiatry, Department of Neuroscience, Yamaguchi University Graduate School of Medicine, Yamaguchi, Japan; 4 Institute of Women’s Health, Tokyo Women’s Medical University, Tokyo, Japan; 5 Department of Psychiatry, Tokyo Metropolitan Tama Medical Center, Tokyo, Japan; University of California, San Francisco, United States of America

## Abstract

**Background:**

On March 11, 2011, Japan was struck by a massive earthquake and tsunami. The tsunami caused tremendous damage and traumatized several people, including children. The aim of this study was to assess changes in traumatic symptoms 8, 20, and 30 months of the 2011 tsunami.

**Methods:**

The study comprised three groups. Copies of the Post-Traumatic Stress Symptoms for Children 15 items (PTSSC-15), a self-rating questionnaire on traumatic symptoms, were distributed to 12,524 children (8-month period), 12,193 children (20-month period), and 11,819 children (30-month period). An effective response of children 8 months, 20 months, and 30 month after the disaster was obtained in 11,639 (92.9%), 10,597 (86.9%), and 10,812 children (91.4%), respectively. We calculated the total score, PTSD subscale, and Depression subscale of PTSSC-15. We calculated the total score, PTSD subscale, and Depression subscale of PTSSC-15.

**Results:**

The PTSSC-15 total score and PTSD subscale of children belonging to 1st–9th grade groups who were tested 30 and 20 months after the tsunami significantly decreased compared with those of children tested 8 months after the tsunami. The PTSSC-15 total score and PTSD subscale of children in 1st–9th grade groups tested after 30 months did not decrease significantly compared with those of children tested after 20 months. The PTSSC-15 Depression subscale and PTSD subscale of children in 1st–9th grade groups tested after 30 months significantly decreased compared with those of children tested 8 months after the tsunami. The PTSSC-15 Depression subscale of children in 1st–9th grade groups evaluated after 30 months significantly decreased compared with those of children evaluated after 20 months.

**Conclusions:**

This study demonstrates that the traumatic symptoms of children who survived the massive tsunami improved with time. Nonetheless, the traumatic symptoms, which in some cases did not improve with time.

## Background

On March 11, 2011, Japan was struck by a huge earthquake and tsunami. Numerous people who survived the tremendous tsunami were traumatized by the miserable experience such as the loss of parents, siblings, and friends [1]–[12]. For all psychiatrists treating the survivors, the post-traumatic stress disorder (PTSD) should be considered most carefully after a disaster [13]–[17]. Many studies have been conducted on children who have survived disasters and had traumatic symptoms [7]–[13], [18]–[30].

Previously, 8 and 20 months after the 2011 Japanese tsunami, we collected information on activities of daily living, damage to environmental conditions, and traumatic symptoms of children who survived the disaster [9]. This research showed that traumatic symptoms depend on gender, age, damage to environmental conditions, and bereavement experience and that the traumatic symptoms and activities of daily living of child survivors improved after 20 months compared with 8 months after the disaster. Furthermore, previous studies showed that traumatic symptoms tend to spontaneously heal over time; therefore, severity of PTSD depends on the time elapsed, individual differences, and methods used in a survey [20], [22], [31]–[36].

Thirty months after the disaster, we collected the same information regarding the traumatic symptoms of child survivors of the 2011 tsunami. It is necessary to test whether the traumatic symptoms of the children who survived the disaster improved 30 months after the tsunami in comparison with the 20-month time point.

The main hypothesis of this study was that traumatic symptoms of children who survived the tsunami improved progressively 8, 20, and 30 months after the disaster. This study aimed to evaluate and compare the changes in the traumatic symptoms of children 20 and 30 months after the 2011 tsunami. Proviso, this survey did not track down the cause of each individual’s traumatic symptoms. Our study shows only the improvement in traumatic symptoms of children in Ishinomaki City.

## Methods

### Study design and settings

This study involved observation of changes in the traumatic symptoms among children after the 2011 Japanese earthquake and tsunami. Ishinomaki City is the second largest city in the Miyagi Prefecture, Japan. Before the disaster, the population of Ishinomaki city was 162,822. As of February 15, 2011, the total number of collapsed houses and buildings, including half-destroyed houses, was 33,378, and 7,298 temporary houses had been constructed. As of April 31, 2014, the death toll in Ishinomaki City was 3,270, and 438 people were missing. After the disaster, 6,583 people moved outside of Ishinomaki city.

The population of Ishinomaki city 8 months, 20 months, and 30 months after the disaster was about 15 million. The number of children 8 months, 20 months, and 30 months after the disaster was 12,524, 12,193, and 11,819 children in municipal schools of Ishinomaki City.

### Recruitment and participants

Each survey was conducted as part of the school education program under the initiative of the Board of Education in Ishinomaki City. Surveys were distributed to all children who attended five kindergartens, 43 elementary schools, and 21 junior high schools in Ishinomaki City, Miyagi Prefecture. The survey was conducted in November of 2011 and 2012 and in September of 2013 (8, 20, and 30 months after the disaster, respectively).

The method of administering the surveys in 2011, 2012, and 2013 was the same. First, the Education Committee of Ishinomaki City explained the survey method to the principals of all schools. Subsequently, the teachers distributed a letter explaining the survey, which had been constructed by the Education Committee, to all children and their parents. The letter clearly stated that by filling out the questionnaire, both the student and parents were consenting to participate in the survey. The letter also specified that the survey results would be used to provide children with psychological care to facilitate their education at the school and that the results would be published as a medical research article. Informed consent was thus obtained when the students filled out the questionnaire. The Ethics Committee of the National Center for Global Health and Medicine approved the survey protocol including the consent procedure.

On November 2011 (8 months after the disaster), the Post-traumatic Stress Symptoms in Children 15 items (PTSSC-15; a self-rating questionnaire on traumatic symptoms) was distributed to 12,524 children enrolled in municipal schools of Ishinomaki City. A questionnaire on the environmental damage experienced by the children was distributed to their teachers.

On November 2012 (20 months after the disaster) and September 2013 (30 months after the disaster), copies of PTSSC-15 were distributed to 12,193 and 11,819 children enrolled in municipal schools and to their teachers.

Parents of kindergartners and 1st–3rd-grade elementary school students were asked to fill out the questionnaire by questioning their children. Informed consent for participation in the survey was obtained when the completed questionnaires were received from the children.

Answers were obtained from 12346 (98.6%) of the 12,524 children (8-month period), 11,124 (91.2%) of the 12,193 children (20-month period), and 11197 (94.7%) of the 11,819 children (30-month period) to whom the questionnaires were sent. An effective response was obtained from 11,639 (92.9% of the 8-month group), 10,597 (86.9% of the 20-month group), and 10,812 children (91.4% of the 30-month group). The effective responses at 30 months had no connection with the effective responses at 20 months because of the anonymity. Similarly, the effective responses at 20 months had no connection with the effective responses at 8 months for the same reason.

Answers to the questionnaire on environmental damage 8 months after the disaster with regard to all 12,524 children were obtained from teachers. Table 1 shows data on gender, age, and damage to environmental conditions (house damage, evacuation conditions, and bereavement experience) for 11,639 children 8 months after the disaster [7]–[12]. When teachers had no information regarding house damage, evacuation conditions, and bereavement experience, the answer was marked “unknown.”

**Table 1:** Damage to the living conditions of children affected by the 2011 Japan earthquake and tsunami.

Items			N = 11,639		
House damage	No		6986	(60.0%)	
	Yes	Total collapse	2243	(19.3%)	
		Half-collapse	2354	(20.2%)	
		Total	4597	(39.5%)	
	Unknown		56	(0.5%)	
Evacuation experience	No		8228	(70.7%)	
	Yes	Currently living in an evacuation center	90	(0.8%)	
		Used to live in an evacuation center	2845	(24.4%)	
		Living in temporary housing	976	(8.4%)	
		Used to live in temporary housing	51	(0.4%)	
		Evacuation experience at least once	3248	(27.9%)	
	Unknown		163	(1.4%)	
Bereavement experience	No		9241	(79.4%)	
	Yes	Father	71	(0.6%)	
		Mother	66	(0.6%)	
		Brothers and sisters	44	(0.4%)	
		Grandfather and grandmother	355	(3.1%)	
		Classmates	1498	(12.9%)	
		Teacher in-charge	32	(0.3%)	
		Others	270	(2.3%)	
		At least one bereavement experience	2103	(18.1%)	
	Unknown		295	(2.5%)	

M, mean; SD, standard deviation; N, number of cases.

### Measures

This was a paper-based survey with questions regarding traumatic symptoms in the self-rating format. The self-rating questionnaire consisted of PTSSC-15.

### PTSSC-15

PTSSC-15 is a self-rating questionnaire on the stress reactions in children after a disaster. Five questions that are believed to reveal important psychosomatic characteristics after a disaster (flashbacks, appetite loss, somatic reactions such as headache and abdominal pain, attention deficit, and anxiety) were added to the Post-Traumatic Stress Symptoms 10-item (PTSS-10) questionnaire that was used as a screening test after the Great Hanshin earthquake and the 2004 Southeast Asia tsunami [14], [37], [38]. PTSSC-15 consisting of 15 questions was constructed in Japan [39]. Each question in the questionnaire is scored on a six-point scale: 0 = completely disagree, 1 = mostly disagree, 2 = partially disagree, 3 = partially agree, 4 = mostly agree, and 5 = completely agree. A high total score indicates more severe traumatic symptoms.

The depression subscale consists of insomnia (Question 1), withdrawal (Question 5), appetite loss (Question 12), inattention (Question 13), and physical symptoms (Question 14). The PTSD subscale consists of irritability (Question 4), displeasure (Question 6), emotional upset (Question 7), avoidance (Question 8), nervousness (Question 9), guilt (Question 10), flashbacks (Question 11), and anxiety (Question 15). Tominaga and colleagues demonstrated reliability and validity of PTSSC-15 in Japanese children and adolescents [40].

### Statistical analysis

Previous studies show that the average total score in PTSSC-15 varies depending on gender and school grade [7], [8], [11], [12]. Therefore, the whole study group of children was subdivided into eight gender and grade groups: male and girl kindergartners, male and girl 1st–3rd-graders (elementary school students), male and girl 4th–6th-graders (elementary school students), and boy and girl 7th–9th-graders (junior high school students).

The average PTSSC-15 total score, Depression subscale, and PTSD subscale in each grade and gender group were calculated separately for the three time points: 8, 20, and 30 months after the tsunami. The differences in the average PTSSC-15 total score, Depression subscale, and PTSD subscale after 8, 20, and 30 months were assessed by two-factor analysis of variance for each gender and period (time point). Furthermore, these differences were compared using Bonferroni post-tests to compare periods. In all tests, a significance level of 0.05 was used in two-sided tests. All calculations were performed using PASW 18.0 and Prism 5.

## Results

### Descriptive information

The participants who were evaluated 8 months after the disaster including 11,639 children (5,939 males and 5,700 girls) and after 20 months including 10,597 children (5,302 boys and 5,295 girls), while those evaluated after 30 months including 10,812 children (5,434 boys and 5,378 girls) who experienced the 2011 Japanese earthquake and tsunami. Table 2 shows the gender, PTSSC-15 total score, Depression subscale, PTSD subscale, and age.

**Table 2:** Characteristics of children affected by the 2011 Japan earthquake and tsunami.

Items		After 8 months		After 20 months		After 30 months	
		N = 11,639		N = 10,597		N = 10,812	
Gender	Boys	5939	(51.0%)	5302	(50.0%)	5434	(50.3%)
	Girls	5700	(49.0%)	5295	(50.0%)	5378	(49.7%)
Age at the time of the disaster (y) (Mean)		10.9	(SD = 2.7)	10.9	(SD = 2.7)	10.9	(SD = 2.7)
PTSSC-15 Total score (Mean)		20.5	(SD = 14.5)	18.8	(SD = 14.0)	19.7	(SD = 14.2)
PTSSC-15 Depression score (Mean)		5.4	(SD = 4.6)	5.0	(SD = 4.4)	4.1	(SD = 3.6)
PTSD score (Mean)		12.6	(SD = 9.1)	11.7	(SD = 8.7)	11.7	(SD = 8.8)

### PTSSC-15 total score after 8, 20, and 30 months

**Figure 1:**
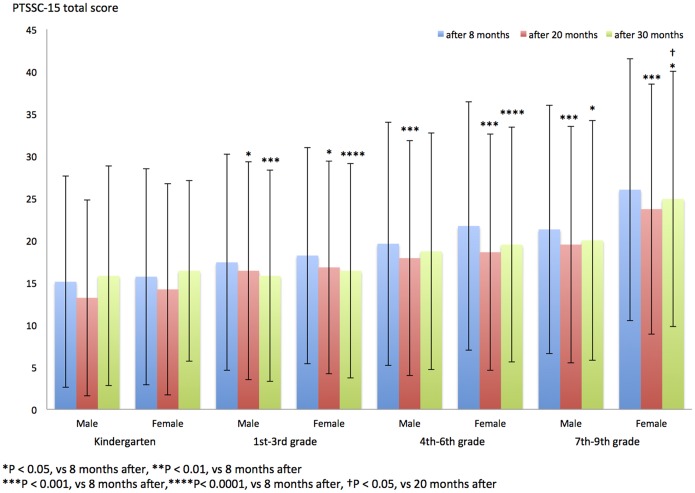
PTSSC-15 total score after 8, 20, and 30 months in each grade group and gender.

The average PTSSC-15 total score was compared in each grade group, gender group, and period group (Table 3, Fig. 1). The PTSSC-15 total score of children in 1st–9th grade groups who were evaluated after 30 months and 20 months significantly decreased compared with that of children tested at 8 months (1st–3rd-grade boys, 20-month period vs 8-month period: P<0.05; 4th–9th-grade boys and 1st–9th-grade girls, 20-month period vs 8-month period: P<0.001; 1st–6th grade, 30-month period vs 8-month period: P<0.001; 7th–9th grade, 30-month period vs 8-month period: P<0.05; Fig. 1).

**Table 3:** Average PTSSC-15 total score (by grade, gender, or period).

Grade group	Gender	Months after disaster												
		2011			2012			2013				F	P value	
		After 8 months			After 20 months			After 30 months						
		M	SD	N	M	SD	N	M	SD	N				
Kindergartners	Boys	15.1	12.5	119	13.2	11.6	111	15.8	13	95	Gender ×Period	0.021	N.S.	
	Girls	15.7	12.8	127	14.2	12.5	127	16.4	10.7	123	Period	2.415	N.S.	
											Gender	0.64	N.S.	
1st–3rd grade	Boys	17.4	12.8	1866	16.4	12.9	1632	15.8	12.5	1592	Gender ×Period	0.212	N.S.	
	Girls	18.2	12.8	1736	16.8	12.6	1581	16.4	12.7	1615	Period	16.18	<0.0001	
											Gender	5.721	<0.05	
4th–6th grade	Boys	19.6	14.4	1975	17.9	13.9	1792	18.7	14	1854	Gender ×Period	2.832	N.S.	
	Girls	21.7	14.7	1973	18.6	14	1798	19.5	13.9	1807	Period	27.5	<0.0001	
											Gender	20.06	<0.0001	
7th–9th grade	Boys	21.3	14.7	1979	19.5	14	1767	20	14.2	1893	Gender ×Period	0.555	N.S.	
	Girls	26	15.5	1864	23.7	14.8	1789	24.9	15.1	1833	Period	18.11	<0.0001	
											Gender	270.9	<0.0001	

M, mean; SD, standard deviation; N, number of cases; N.S., not significant.

The PTSSC-15 total score of children in 1st–9th grade groups who were tested after 30 months did not significantly decrease compared with that of children tested at 20 months (Fig. 1).

### PTSSC-15 Depression subscale after 8, 20, and 30 months

**Figure 2:**
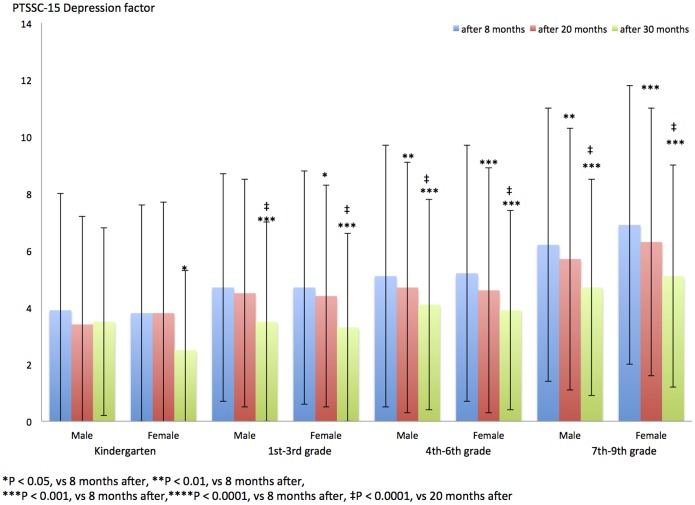
PTSSC-15 Depression factor after 8, 20, and 30 months in each grade group and gender.

Table 4 and Fig. 2 show the PTSSC-15 Depression score after 8, 20, and 30 months in each grade and gender group. The PTSSC-15 Depression subscale and PTSD subscale of children in 1st–9th-grade groups who were tested after 30 months significantly decreased compared with those of children tested at 8 months (1st–9th grades, 30-month period vs 8-month period: P<0.001; Fig. 2).

**Table 4:** Average score on the PTSSC-15 depression subscale (by grade, gender, or period).

Grade group	Gender	Months after disaster											
		2011 After 8 months			2012 After 20 months			2013 After 30 months				F	P value
		M	SD	N	M	SD	N	M	SD	N			
Kindergartners	Boys	3.9	4.1	119	3.4	3.8	111	3.5	3.3	95	Gender × Period	2.184	N.S.
	Girls	3.8	3.8	127	3.8	3.9	127	2.5	2.8	123	Period	3.312	<0.05
											Gender	0.709	N.S.
1st–3rd grade	Boys	4.7	4.0	1866	4.5	4.0	1632	3.5	3.5	1592	Gender × Period	0.570	N.S.
	Girls	4.7	4.1	1736	4.4	3.9	1581	3.3	3.3	1615	Period	108.5	<0.0001
											Gender	1.711	N.S.
4th–6th grade	Boys	5.1	4.6	1975	4.7	4.4	1792	4.1	3.7	1854	Gender × Period	1.235	N.S.
	Girls	5.2	4.5	1973	4.6	4.3	1798	3.9	3.5	1807	Period	70.37	<0.0001
											Gender	0.705	N.S.
7th–9th grade	Boys	6.2	4.8	1979	5.7	4.6	1767	4.7	3.8	1893	Gender × Period	1.080	N.S.
	Girls	6.9	4.9	1864	6.3	4.7	1789	5.1	3.9	1833	Period	130.7	<0.0001
											Gender	44.60	<0.0001

M, mean; SD, standard deviation; N, number of cases; N.S., not significant.

The PTSSC-15 Depression subscale and PTSD subscale of children in the kindergarten and 1st–3th grades (girls) and in 4th–9th-grade groups who were tested after 20 months significantly decreased compared with those of children tested at 8 months (kindergarteners and 1st–3rd-grade girls, 20-month period vs 8-month period: P<0.05; 4th–9th-grade boys, 20-month period vs 8-month period: P<0.01; 4th–9th-grade girls, 20-month period vs 8-months period: P<0.001; Fig. 2).

The PTSSC-15 Depression subscale of children in 1st–9th grade groups who were tested after 30 months significantly decreased compared with that of children evaluated at 20 months (kindergarten girls, 20-month period vs 8-month period: P<0.05; 1st–9th grades, 20-month period vs 8-months period: P<0.001, Fig. 2).

### PTSSC-15 PTSD score after 8, 20, and 30 months

**Figure 3:**
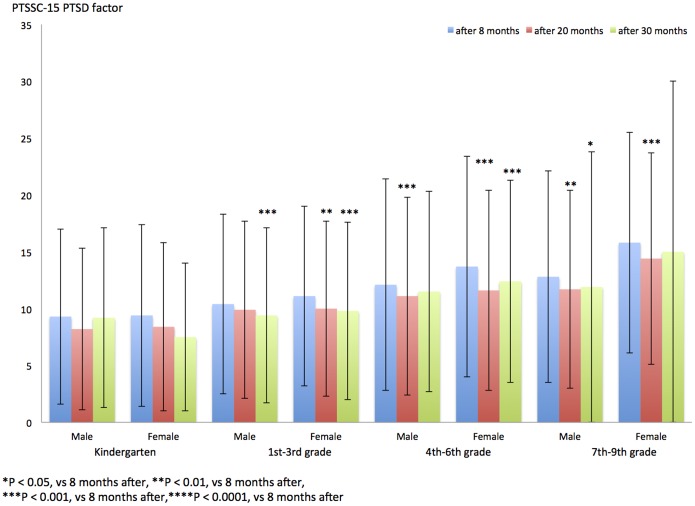
PTSSC-15 PTSD factor after 8, 20, and 30 months in each grade group and gender.

Table 5 and Fig. 3 show the PTSSC-15 PTSD subscale after 8, 20, and 30 months in each grade and gender group. The PTSSC-15 PTSD subscale of children in 1st–9th grade groups who were tested after 30 months and 20 months significantly decreased compared with that of children tested at 8 months (1st–3rd-grade girls and 4th–9th-grade boys, 20-month period vs 8-month period: P<0.01; 4th–9th-grade girls, 20-month period vs 8-month period: P<0.001; 1st–3rd grades and 4th–6th-grade girls, 30-month period vs 8-month period: P<0.001; 7th–9th-grade boys, 30-month period vs 8-month period: P<0.05; Fig. 3). The PTSSC-15 PTSD subscale of children in 1st–9th grade groups who were evaluated after 30 months did not significantly decrease compared to that of children tested at 20 months (Fig. 3).

**Table 5:** Average PTSSC-15 PTSD score (by grade, gender, or period).

Grade group	Gender	Months after disaster											
		2011 After 8 months			2012 After 20 months			2013 After 30 months				F	P value
		M	SD	N	M	SD	N	M	SD	N			
Kindergartners	Boys	9.3	7.7	119	8.2	7.1	111	9.2	7.9	95	Gender × Period	1.196	N.S.
	Girls	9.4	8.0	127	8.4	7.4	127	7.5	6.5	123	Period	1.468	N.S.
											Gender	0.683	N.S.
1st–3rd grade	Boys	10.4	7.9	1866	9.9	7.8	1632	9.4	7.7	1592	Gender × Period	1.230	N.S.
	Girls	11.1	7.9	1736	10.0	7.7	1581	9.8	7.8	1615	Period	18.99	<0.0001
											Gender	6.558	N.S.
4th–6th grade	Boys	12.1	9.3	1975	11.1	8.7	1792	11.5	8.8	1854	Gender × Period	3.523	<0.05
	Girls	13.7	9.7	1973	11.6	8.8	1798	12.4	8.9	1807	Period	27.77	<0.0001
											Gender	34.09	<0.0001
7th–9th grade	Boys	12.8	9.3	1979	11.7	8.7	1767	11.9	11.9	1893	Gender × Period	0.339	N.S.
	Girls	15.8	9.7	1864	14.4	9.3	1789	15.0	15.0	1833	Period	12.76	<0.0001
											Gender	202.1	<0.0001

M, mean; SD, standard deviation; N, number of cases; N.S., not significant.

## Discussion

This study shows that the PTSSC-15 total score, PTSD subscale, and Depression subscale in child survivors changed with time after this severe natural disaster. Other studies showed that the PTSSC-15 total score, PTSD subscale, and Depression subscale are associated with the environmental damage caused by the 2011 Japanese tsunami [7], [8], [11], [12]. Some other studies showed that the PTSSC-15 total score 20 months after the tsunami improved compared with that at the 8-month time point [9]. In the elementary school children, the mean total PTSSC-15 score, PTSSC-15 PTSD score, and PTSSC-15 Depression score significantly improved 20 months after the tsunami compared with that at the 8-month period, whereas no significant improvements were observed in the junior high school students [7], [8], [11], [12].

The present study shows that the traumatic and depressive symptoms of children who survived the 2011 tsunami did not improve progressively 8, 20, and 30 months after the disaster. Our results demonstrate that traumatic symptoms of elementary school and junior high school students improved at 20 months compared with those at the time point 8 months after the disaster. Nonetheless, traumatic symptoms and PTSD symptoms of elementary school and junior high school students at 30 months did not improve compared to those at the 20-month period. On the other hand, depression symptoms of elementary school and junior high school students improved at 20 months compared with those at 8 months after the 2011 tsunami. Depression symptoms of elementary school and junior high school students after 30 months improved compared with those at the 20-month period. These results indicate that the PTSD subscale of traumatic symptoms of the children survivors probably improved only up to 20 months after the massive disaster. Therefore, the main hypothesis of this study was refuted. The changes in the PTSD subscale and Depression subscale of traumatic symptoms by gender, age, and time points were found in this and previous studies.

This study demonstrates that the mental state of kindergarten children did not significantly improve with time. The total score, PTSD subscale, and Depression subscale were calculated from PTSSC-15 filled by the parents. The stage of language development was probably not mature enough in this group of children to answer the questions related to traumatic symptoms. It seems that they could not understand the questionnaires about traumatic symptoms and could not answer these questions using adequate words. Accordingly, clinicians treating kindergartener survivors evaluate their psychiatric symptoms not only using questionnaires but also by observing the activities of daily living such as playing with friends and the mother-child relationship.

Clinically, it is important that children’s traumatic symptoms after a huge disaster be subdivided into PTSD symptoms and depression symptoms. Additionally, psychiatrists should not forget to take into account the age of a child. According to our results, the depressive symptoms of children survivors of the massive disaster improved over time. In contrast, PTSD symptoms of these children improved only up to 20 months after the event. It is difficult to assess traumatic symptoms of kindergarteners accurately. Therefore, it is necessary to comprehensively assess the status of children by considering not only their traumatic symptoms including PTSD and depression symptoms but also their age, gender, and the time elapsed after a disaster.

## Limitations

This survey had two limitations in the methodology. This survey was conducted only at three time points: 8 months, 20 months, and 30 months after the 2011 tsunami. Furthermore, this survey was based on a self-rating questionnaire and was conducted in only one district in Japan. It is impossible to determine when the traumatic symptoms stopped decreasing: before or after the 20-month time point. It is also impossible to calculate severity of PTSD in children after the 2011 Japanese earthquake and tsunami based on the results of our survey.

Another limitation is that this survey did not track down the cause of each individual’s traumatic symptoms. Our study shows only the improvement in traumatic symptoms of children in Ishinomaki City. Therefore, this study cannot serve as an epidemiological survey or cohort study for formulating a psychiatric diagnosis. Examination by child psychiatrists using operational diagnostic criteria and structured interviews is still necessary for accurate psychiatric diagnosis. Moreover, the results of this study on children in Ishinomaki City do not reflect all the characteristics of children affected by the 2011 Japanese earthquake and tsunami.

## Conclusion

This study demonstrates that the traumatic symptoms of children who survived the massive tsunami improved with time. Nonetheless, the traumatic symptoms including PTSD and depressive symptoms did not improve consistently 8, 20, and 30 months after the disaster. Thus, it is important not only to evaluate the traumatic symptoms using a self-rating questionnaire but also to analyze specific information regarding PTSD symptoms, depression symptoms, age, gender, and the time elapsed after a disaster.
